# Active interoceptive inference and the emotional brain

**DOI:** 10.1098/rstb.2016.0007

**Published:** 2016-11-19

**Authors:** Anil K. Seth, Karl J. Friston

**Affiliations:** 1 Sackler Centre for Consciousness Science, School of Engineering and Informatics, University of Sussex, Falmer, Brighton BN1 9QJ, UK; 2 Wellcome Trust Centre for Neuroimaging, Institute of Neurology, UCL, London WC1N 3BG, UK

**Keywords:** emotion, self, interoception, predictive coding, neuromodulation, cybernetics

## Abstract

We review a recent shift in conceptions of interoception and its relationship to hierarchical inference in the brain. The notion of interoceptive inference means that bodily states are regulated by autonomic reflexes that are enslaved by descending predictions from deep generative models of our internal and external milieu. This re-conceptualization illuminates several issues in cognitive and clinical neuroscience with implications for experiences of selfhood and emotion. We first contextualize interoception in terms of active (Bayesian) inference in the brain, highlighting its enactivist (embodied) aspects. We then consider the key role of uncertainty or precision and how this might translate into neuromodulation. We next examine the implications for understanding the functional anatomy of the emotional brain, surveying recent observations on agranular cortex. Finally, we turn to theoretical issues, namely, the role of interoception in shaping a sense of embodied self and feelings. We will draw links between physiological homoeostasis and allostasis, early cybernetic ideas of predictive control and hierarchical generative models in predictive processing. The explanatory scope of interoceptive inference ranges from explanations for autism and depression, through to consciousness. We offer a brief survey of these exciting developments.

This article is part of the themed issue ‘Interoception beyond homeostasis: affect, cognition and mental health’.

## 1. Introduction

Recent years have seen the emergence of a framework within cognitive neuroscience that offers exactly the right set of concepts to talk about the body and mind in terms of beliefs about the body (and oneself). On this view, the brain is not an elaborate stimulus-response link but a statistical organ that actively generates explanations for the stimuli it encounters—in terms of hypotheses that are tested against sensory evidence. This perspective can be traced back to Helmholtzian formulations of unconscious inference [1]. Over the last few years, the underlying idea has been formalized to cover deep or hierarchical Bayesian inference–about the hidden causes of our sensations—and how these inferences induce beliefs and behaviour [2–7]. ‘Explanations’, ‘hypotheses’ and ‘beliefs’ should in this context be understood not as consciously held mental states, but as neuronally encoded probability distributions (i.e. Bayesian beliefs) over the hidden causes of sensory signals. The biophysical encoding of these ‘beliefs’ is, technically, in terms of sufficient statistics like the mean or expectation of a distribution.

**Figure 1.**
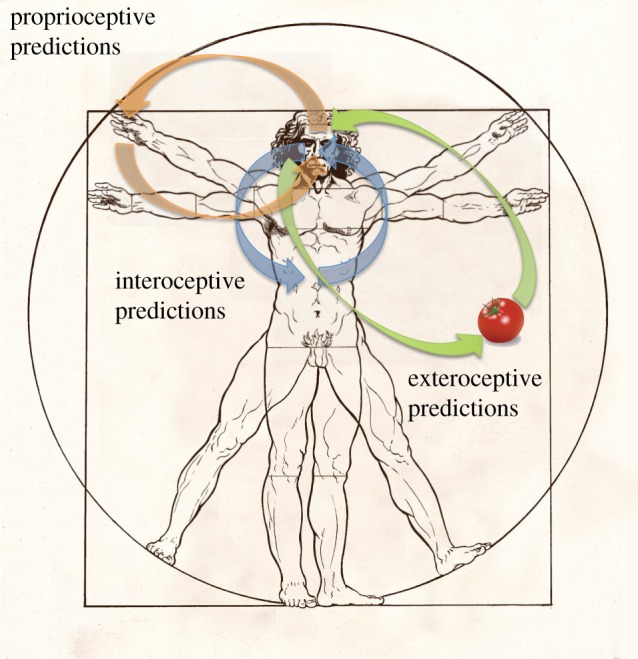
Inference and perception across different modalities. Green arrows represent exteroceptive predictions and prediction errors underlying perception of the external world. Orange arrows represent proprioceptive predictions (and prediction errors) generating action through active inference. Blue arrows represent interoceptive predictions (and prediction errors) underlying emotional processing and autonomic regulation. Integrated experiences of embodied selfhood emerge from the joint hierarchical content of self-related predictions across all these dimensions, including—at hierarchically deep levels—multimodal and amodal predictions. Adapted from Seth [7]. (Online version in colour.)

In the last few years, ‘Bayesian brain’ ideas have been applied in the context of *interoception* (figure 1), which refers to the perception and integration of autonomic, hormonal, visceral and immunological signals [8,9]—or more informally as the sense of the body ‘from within’. On some of these views [7,10], emotional experience and experiences of embodied selfhood emerge from top-down inference on the (multimodal) causes of interoceptive afferents, generalizing so-called two-factor or evaluative theories of emotion and cognition [11]. A first implication of these proposals is that these kinds of perceptual experience are as subject to (implicit and perhaps idiosyncratic) beliefs, as are perceptions of the external world. Beyond this, the context of interoception brings about further shifts in how to think about the relationships between body, mind and brain. One such shift is that generative models of interoceptive signals should be geared towards *control* or *regulation* of physiological variables, rather than towards accurate representation of some extra-cranial state-of-affairs [7,12,13]. The two goals remain tightly interwoven, inasmuch as effective regulation depends on deployment of sufficiently elaborated predictive models. This shift recognizes alternative origins to ‘Bayesian brain’ ideas in twentieth century ‘cybernetics’ [14,15] and in doing so, points to a deep connection between *life* and *mind*, in which cognitive processes are grounded in fundamental evolutionary imperatives to maintain physiological homeostasis [7,16]. Many other specific implications follow, for example in reframing the functional basis of a variety of disorders of emotion and selfhood.

Here, we survey these exciting developments. We first provide a brief introduction to the framework of prediction error minimization in the Bayesian brain, emphasizing its embodied or enactive aspects. These aspects appear prominently in the role of action in reducing prediction error (i.e. active inference) and emphasize the key role of uncertainty or precision in shaping the interplay between prior beliefs and sensory evidence. Precision-weighting of recurrent signalling in cortical hierarchies is closely associated with neuromodulation, providing important clues about developmental origins of conditions like autism; it is also associated with attention, suggesting novel accounts for symptom expression due to aberrant attention to interoceptive signals.

Turning to functional neuroanatomy, we outline the functional architecture of interoceptive inference and review recent suggestions that perceptual predictions originate preferentially in agranular cortices [9,17], while acknowledging that direct empirical evidence for interoceptive inference is still to be uncovered. We next address some theoretical issues, relating active interoceptive inference to experiences of emotion and embodied selfhood, highlighting a control-oriented or instrumental perspective on interoceptive inference that calls on cybernetic concepts of predictive regulation, allostatic control and perceptual control theory [7,13,18]. We conclude by exploring the implications of these ideas for a sample of clinical conditions that may reflect false interoceptive inference, either in their aetiology and/or in symptom expression. While disorders in emotional processing and interoceptive experience naturally invite explanations in terms of abnormal interoceptive inference, we also highlight how this perspective can illuminate other conditions and symptoms including autism, fatigue and depression.

## 2. Predictive coding in the Bayesian brain

Current formulations of Helmholtz's notion are now the most popular metaphors for neuronal processing and are usually considered under the Bayesian brain hypothesis as predictive coding [6,19–21]. Predictive coding is a process theory with a biologically plausible back story and a considerable amount of empirical support [21,22]. (See [23] for a review of canonical microcircuits and predictive coding in perception, [17,24] for an application of the same ideas to motor control, and [25] for evidence of feed-forward and feed-back signalling carried by distinct frequency bands.)

**Figure 2.**
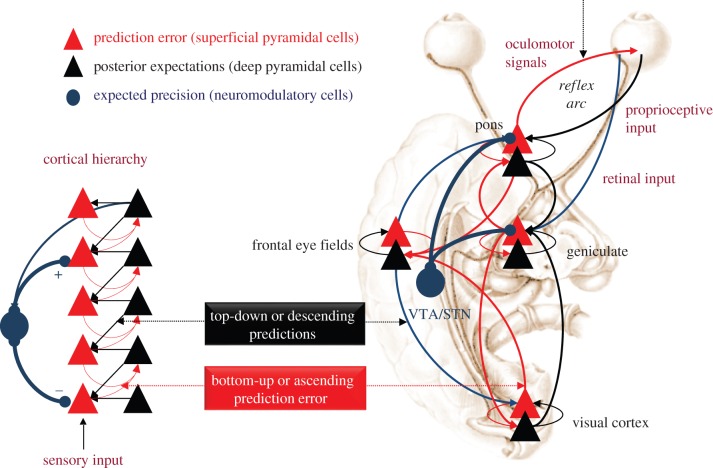
This figure summarizes the hierarchical neuronal message passing that underlies predictive coding. The basic idea is that neuronal activity encodes *expectations* about the causes of sensory input, where these expectations minimize prediction *error*. Prediction error is the difference between (ascending) sensory input and (descending) predictions of that input. This minimization rests upon recurrent neuronal interactions between different levels of the cortical hierarchy. Current interpretations suggest that superficial pyramidal cells (red triangles) compare the expectations (at each level) with top-down predictions from deep pyramidal cells (black triangles) of higher levels [22,23]. On the left: this schematic shows a simple cortical hierarchy with ascending prediction errors and descending predictions. This graphic includes neuromodulatory gating or gain control (blue) of superficial pyramidal cells that determines their relative influence on deep pyramidal cells encoding expectations through modulation of expected precision (see below and text for details). On the right: this provides a schematic example in the visual system. It shows the putative cells of origin of ascending or forward connections that convey prediction errors (red arrows) and descending or backward connections (black arrows) that construct predictions. The prediction errors are weighted by their expected precision that we have associated with the activity of neuromodulatory systems—here projections from ventral tegmental area (VTA) and substantia nigra (STN). In this example, the frontal eye fields send predictions to primary visual cortex, which it projects to the lateral geniculate body. However, the frontal eye fields also send proprioceptive predictions to pontine nuclei, which are passed to the oculomotor system to cause movement through classical reflexes. These descending predictions are also passed to the lateral geniculate body and constitute corollary discharge. Every top-down prediction is reciprocated with a bottom-up prediction error to ensure predictions are constrained by sensory information. The resolution of *proprioceptive* prediction error is particularly important because this enables descending predictions—about the state of the body—to cause movement by dynamically resetting the equilibrium or set-point of classical reflexes. Resolving sensory prediction errors through action is known as *active inference* (see the text). Adapted from Friston [26]. (Online version in colour.)

**Figure 3.**
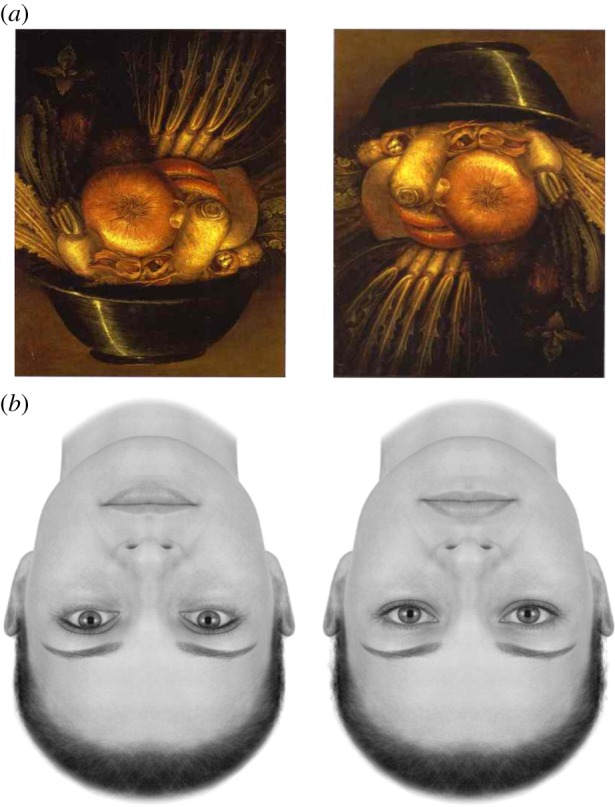
(*a*) Giuseppe Arcimboldo, The Vegetable Gardener (*ca* 1590). Oil on panel. Our percepts are constrained by what we expect to see and the hypotheses that can be called upon to explain sensory input [27]. Arcimboldo, ‘a 16th century Milanese artist who was a favourite of the Viennese, illustrates this dramatically by using fruits and vegetables to create faces in his paintings. When viewed right side up, the paintings are readily recognisable faces’ [28, p. 204]. Adapted from Friston [26]. (*b*) Faces are probably one of the most important (hidden) causes of our sensations. While in Arcimboldo's image, viewing right side up is needed for the configuration of features to appear as a face, when images are already recognizable faces, viewing right side up (by rotating the page) reveals that these faces might in fact be more different than they appear (this is the so-called ‘Thatcher illusion’). These examples illustrate the complex interplay between prior expectations and stimulus features that shape perceptual content (adapted from Little *et al.* [29]). (Online version in colour.)

In these schemes, neuronal representations in higher or deeper levels of neuronal hierarchies generate predictions of representations in lower levels. These descending predictions are compared with lower-level representations to form a prediction error (usually associated with the activity of superficial pyramidal cells). This mismatch or difference signal is passed back up the hierarchy, to update higher representations (usually associated with the activity of deep pyramidal cells). The recurrent exchange of signals between adjacent hierarchical levels resolves prediction error at each and every level, resulting in a hierarchically deep explanation for sensory inputs. In computational terms, the activity of neuronal populations is assumed to encode Bayesian beliefs or probability distributions over states in the world that cause sensations (e.g. my visual sensations are caused by a *face*—see figures 2 and 3). The simplest encoding corresponds to representing the belief with the expected value (mean) of a (hidden) cause or *expectation*. These causes are referred to as *hidden* because they have to be inferred from their sensory consequences. In other words, they can never be directly observed and are forever hidden behind a sensory veil.

In short, predictive coding represents a biologically plausible scheme for updating beliefs about the world based on sensory samples (figure 2). In this setting, neuroanatomy and neurophysiology can be regarded as a distillation of statistical or causal structure in the environment that is disclosed by sensory samples. The resulting anatomy of connections and their physiology furnish a generative model—generating predictions of sensations that can be compared with actual sensory samples. Empirical evidence is now emerging that shows how prior expectations shape behavioural and neuronal signatures of perception, with recent studies in vision [30–32] and audition [33] providing excellent examples. More generally, this view of perception emphasizes ‘the beholder's share’. See also figure 3:

The insight that the beholder's perception involves a top-down inference convinced [the art historian Ernst] Gombrich that there is no ‘innocent eye’: that is, all visual perception is based on classifying concepts and interpreting visual information. One cannot perceive that which one cannot classify. [28, p. 287]

### (a) Embodied (*active*) inference and precision-weighting

There are two key ways in which prediction errors can be reduced: the first is by updating predictions to make them more like the expectations at lower levels (and sensations) currently in play. This process corresponds to perception, as implemented in predictive coding. The second way to resolve prediction errors is to change the sensory samples to make them more like predictions. This entails an active sampling of the sensorium through a redeployment of sensory surfaces: e.g. saccadic eye searches or other sensory palpitations. Placing predictive coding in an embodied or enactive framework in which both action and perception are in the game of minimizing the same prediction error is known as *active inference* [34]. To fully appreciate the bilateral nature of active inference, one has to consider the embodied context in which predictions are made (and fulfilled). These predictions are not only about the world, but also about the body. In brief, perception can be understood as resolving (exteroceptive) prediction errors by selecting predictions that best explain sensations, while behaviour suppresses (proprioceptive) prediction error by changing (proprioceptive) sensations. This suppression rests on classical reflexes, whose equilibrium points are set by descending proprioceptive predictions [24]. For example, an intended movement can be elicited by simply predicting the proprioceptive consequences of a particular movement trajectory, which will be fulfilled by peripheral reflexes. Note that only proprioceptive prediction errors are minimized (at the level of the spinal cord); however, with a good generative model, these movements will also fulfil visual and other exteroceptive (e.g. somatosensory) predictions. This follows because descending (multimodal) predictions emanate from a deep generative model that effectively assimilates prediction errors from all modalities—including interoception. In this context, an important and sometimes overlooked aspect of active inference is that it implies a counterfactual or conditional aspect. That is, in order for an action successfully to reduce prediction error, the brain must represent not only the hidden causes of current sensory signals but also must use these representations to predict how sensory signals would change under specific actions [35]. Interestingly, it has been suggested that such counterfactual aspects of perceptual prediction may underlie basic properties perceptual experience, such as ‘presence’ or ‘objecthood’ [36].

To enable predictions about the consequences of action to be fulfilled, we have to attenuate proprioceptive prediction errors—that would otherwise deliver unequivocal evidence that we are not, in fact, acting. This attenuation rests on reducing the *precision* of proprioceptive prediction errors. Precision can be regarded as a measure of signal-to-noise or confidence. Mathematically, precision is the inverse variance or reliability of a signal. Estimating precision speaks to a fundamental aspect of inference, namely, the encoding of precision or expected uncertainty [37–39]. In other words, we have to infer both the *cause* of our sensations and the *context*, in terms of the (expected or subjective) precision of sensory evidence. This represents a subtle but ubiquitous problem for the brain, where the solution rests on modulating the gain or excitability of neuronal populations reporting prediction error [21,40,41].

Heuristically, one can regard ascending prediction errors in cortical hierarchies as broadcasting ‘newsworthy’ information that cannot be explained by descending predictions. However, the brain also has to select the prediction errors it attends to. It can do this by adjusting their volume or *gain*. Those prediction errors that have been assigned high precision therefore have privileged access to high levels of the hierarchy and can therefore update high-level expectations. Empirical evidence suggests that this precision-weighting is a generic computational process throughout the brain [39] and may be instantiated through neuromodulatory mechanisms of gain control at a synaptic level [42]. The ensuing neuromodulatory gain control corresponds to a (Bayes-optimal) encoding of precision in terms of the excitability of neuronal populations reporting prediction errors. This may explain why superficial pyramidal cells are equipped with so many synaptic gain control mechanisms, such as NMDA receptors and classical neuromodulatory receptors like D1 dopamine receptors [43–46]. Furthermore, it places excitation-inhibition balance in a perfect position to mediate Bayesian belief updating within and among hierarchical levels [47]. This contextual aspect of predictive coding has been associated with attentional gain control in sensory processing [40,48] and has been discussed in terms of affordance in the setting of action selection [49–51]. Crucially, the delicate balance of precision over hierarchical levels can have a profound effect on inference and may underlie false beliefs in psychopathology [52].

## 3. Interoceptive inference

Key challenges for formal accounts of brain function are emotion, self-awareness and their disorders. Recently, people have started to cast emotional processing in terms of predictive coding or inference about interoceptive or bodily states [9,10,53,54]. The basic argument follows the explanation for action above, namely, motor reflexes are driven by proprioceptive prediction errors. Proprioceptive prediction errors compare primary afferents from stretch receptors with proprioceptive predictions that descend to alpha motor neurons in the spinal cord and cranial nerve nuclei. This effectively replaces descending motor commands with proprioceptive predictions, which are fulfilled by peripheral reflexes [24]. These predictions rest on deep hierarchical inference about states of the world, including our own body. Replacing *proprioceptive* signals with *interoceptive* signals, one can see how autonomic reflexes can transcribe descending interoceptive predictions into physiological homoeostasis (e.g. blood pressure, glycaemia, etc.). Importantly, interoceptive predictions constitute just one stream of multimodal predictions that are generated by expectations about the embodied self. On this view, interoceptive signals do not cause emotional awareness, or vice versa. Instead, there is a circular causality, where neuronally encoded predictions about bodily states engage autonomic reflexes through active inference (see below), while interoceptive signals inform and update these predictions. Emotion or affective content then becomes an attribute of any representation that generates interoceptive predictions—where interoception is necessarily contextualized by concurrent exteroceptive and proprioceptive cues (figure 1).

A useful way to think about interoceptive inference is as generalizing physiological (James–Lange) and two-factor or appraisal (e.g. [11]) approaches to emotion. These formulations regard emotional experience as arising from cognitively contextualized perception of changes in bodily state. Interoceptive inference extends these early ideas to incorporate a smooth hierarchy of (precision-weighted) predictions and prediction errors, without assuming any bright line distinction between cognitive and non-cognitive processing. By analogy with predictive coding approaches to visual perception, we propose that emotional content is determined by beliefs (i.e. posterior expectations) about the causes of interoceptive signals across multiple hierarchical levels. An important challenge in this context is to identify which aspects of inference support specifically *conscious* emotional experience, with predictions (rather than prediction errors) being the preferred vehicle [32]. It is tempting to speculate that deep expectations at higher levels of the neuronal hierarchy are candidates for—or correlates of—conscious experience, largely because their predictions are domain general and can therefore be articulated (through autonomic or motor reflexes).

Crucially, interoceptive inference augments appraisal theories with the concept of *active inference*, by which interoceptive predictions can perform physiological homoeostasis by enlisting autonomic reflexes [10,13]. More specifically, descending predictions provide a homoeostatic set-point against which primary (interoceptive) afferents can be compared. The resulting prediction error then drives sympathetic or parasympathetic effector systems to ensure homoeostasis or allostasis, for example, sympathetic smooth-muscle vasodilatation as a reflexive response to the predicted interoceptive consequences of ‘blushing with embarrassment’. This formulation of autonomic reflexes follows exactly the active inference formulation of motor reflexes that enable the contraction of striated muscle to be prescribed or enslaved by equilibrium points set by descending projections to alpha motoneurons in the spinal cord [24].

Active inference highlights a shift from predictive models underlying perception of hidden causes of sensory data, to their use in control or regulation of these causes [7]. Importantly, both (predictive) perception and (predictive) regulation can involve action, as emphasized by distinguishing *epistemic* and *instrumental* active inference [7,12]. The basic idea is that epistemic (active) inference involves selecting actions that we expect to increase the fit between predictive models and hidden causes of sensory signals. This form of inference may characterize, for example, saccadic eye movements [35] or exploratory body movements to inform self-models [55]. Instrumental active inference, by contrast, leverages predictive models to achieve *control* of sensory variables. This perspective has been applied to exteroception in the guise of ‘perceptual control theory’ [18] which emphasizes that ‘control systems control what they *sense*, not what they *do*’ (italics in the original). Instrumental or control-oriented inference is however particularly relevant to interoception, where maintenance of physiological variables within homoeostatically viable ranges is critical to organism survival. In this context, exploratory or epistemic interoceptive ‘actions’ may be less evident because they may be more costly: one does not want to raise one's blood pressure to physiologically dangerous levels just to see whether it can return. The association of predictive models with control of sensory variables recalls the cybernetic view that ‘every good regulator of a system must be a model of that system’ [14, p. 89], and the distinction between instrumental and epistemic actions also highlights the counterfactual aspects of active inference, where potential actions are associated with their likely sensory consequences [7,35,36].

In terms of predictive coding, the balance between homoeostatic reflexes and more goal-directed allostatic behaviour rests upon the confidence (i.e. precision) placed in deeper expectations about how we will behave. For example, hypoglycaemia could induce low-level predictions that mobilize glucose stores (through autonomic reflexes driven by precise interoceptive prediction errors). Alternatively, if we can attenuate the precision of low-level interoception, then proprioceptive predictions can be fulfilled that preclude domain specific homoeostatic responses and engage allostatic behaviour, i.e. preparing and consuming a meal.

As yet, direct empirical evidence for (or against) interoceptive predictions or prediction errors is still lacking. While there is ample circumstantial that fits comfortably with this framework (see [9,10,54,56], for reviews), the principles of interoceptive inference rest primarily on the view that perceptual inference—whether about the world or about the body—is likely to involve a common computational architecture. Moreover, the neuroanatomical properties of brain regions involved in interoceptive processing can be informatively interpreted from this perspective, as we describe next.

### (a) Functional neuroanatomy of interoceptive inference

**Figure 4.**
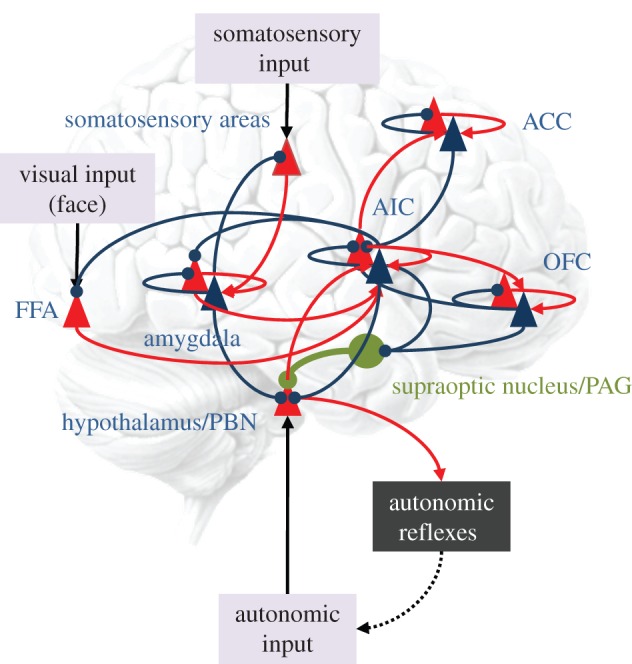
A (simplified) neural architecture underlying the predictive coding of visual, somatosensory and interoceptive signals. The anatomical designations, although plausible, are used to simply illustrate how predictive coding can be mapped onto neuronal systems. As in figure 2, red triangles correspond to neuronal populations (superficial pyramidal cells) encoding prediction error, while blue triangles represent populations (deep pyramidal cells) encoding expectations. These provide descending predictions to prediction error populations in lower hierarchical levels (blue connections). The prediction error populations then reciprocate ascending prediction errors to adjust the expectations (red connections). Arrows denote excitatory connections, while circles denote inhibitory effects (mediated by inhibitory interneurons). In this example, recurrent connections mediate innate (epigenetically specified) reflexes—such as the suckling reflex—that elicit autonomic (e.g. vasovagal) reflexes in response to appropriate somatosensory input. These reflexes depend upon high-level representations predicting both the somatosensory input and interoceptive consequences. The representations are activated by somatosensory prediction errors and send interoceptive predictions to the hypothalamic area—to elicit interoceptive prediction errors that are resolved in the periphery by autonomic reflexes. Oxytocin (in green) is shown to project to the hypothalamic area, to modulate the gain or precision of interoceptive prediction error units. One hypothesis for autism rests on a failure to attenuate the precision of autonomic prediction errors, thereby precluding expectations about visual and somatosensory information (e.g. a mother's face or affiliative touch) that is not accompanied by autonomic input (see the text). FFA, fusiform face area; AIC, anterior insular cortex; ACC, anterior cingulate cortex; OFC, orbitofrontal cortex; PAG, periaqueductal grey; PBN, parabrachial nucleus. (Online version in colour.)

Translating the computational machinery of interoceptive inference into a deeper understanding of brain function requires mapping its computational elements onto neuroanatomical substrates. A number of recent proposals suggest several convergent features [9,10,13]. The first is that so-called visceromotor areas (VMAs), such as the anterior insula cortex (AIC), anterior cingulate cortex (ACC), subgenual cortex (SGC), and perhaps also, orbitofrontal cortex (OFC) are situated at the top of an interoceptive hierarchy. The second is that these areas collectively embody a generative model of interoceptive responses and issue predictions that, when unpacked at the lowest hierarchical level, serve as homoeostatic set-points. These VMAs are known to receive ascending projections from viscerosensory areas (e.g. posterior and mid-insula) and their descending connections engage a range of subcortical, brainstem and spinal cord targets involved in visceromotor control, such as the periaqueductal grey (PAG) and the parabrachial nucleus (PBN) [8,57–59]. Visceromotor efferents also directly innervate viscerosensory areas, potentially providing a form of efference copy or corollary discharge (i.e. descending predictions) enabling the formation of (ascending) interoceptive prediction errors. As well as known anatomical connectivity patterns, this basic architecture is supported by cytoarchitectonic observations that VMAs lack a well formed (granular) layer IV as a target for ascending prediction errors [9]. Such agranular cortical regions are argued to be well suited to the issuing of predictions, in both interoceptive [9] and motor [17] domains. Figure 4 shows a schematic of the sort of functional anatomy implied by interoceptive inference (that we will appeal to later in the context of autism).

### (b) Interoceptive inference and embodied selfhood

Having described the computational architecture of interoceptive inference and its potential functional neuroanatomy, we are now in a position to explore how this framework can illuminate more theoretical issues in the nature and experience of selfhood. In everyday life, we experience our ‘selfhood’ as continuous and integrated. While it may be adaptive to experience being a ‘self’ in this way, it would be a mistake to assume on this basis that there is such a thing as unitary self-process underlying these experiences. Clinical conditions and experimental manipulations amply illustrate that experiences of selfhood unfold across many partially independent and partially overlapping levels of description; levels which can be teased apart in the laboratory or which may fall apart during psychiatric or neurological illness. A simple classification, from ‘low’ to ‘high’ levels, would range from experiences of being and having a body [10,60,61], through to the experience of perceiving the world from a particular point of view (a first person perspective, see [62,63]), to experiences of intention and agency [64,65], and at higher levels the experience of being a continuous self over time (a ‘narrative’ self or ‘I’ that depends on episodic autobiographical memory, see [66]) and finally, a social self, in which my experience of being ‘me’ is shaped by how I perceive others’ perceptions of me [67]. In this putative classification, interoception plays a key role in structuring experiences of ‘being and having a body’ (i.e. embodied selfhood) and may also shape selfhood at other, hierarchically higher levels.

There is accumulating evidence that interoception plays a key role in shaping experiences of body ownership. Illusions of body ownership, like the rubber hand illusion and the so-called ‘full body’ illusion, while normally induced by false visuo-tactile congruence, can also be induced by ‘cardio-visual’ feedback in which a virtual body (or body part) flashes in time with a participant's heartbeat [68,69]. Recent extensions of these studies have also shown that visual feedback of respiratory patterns can have a similar effect [70], providing support for a multimodal influence of interoception on embodied selfhood. The ways in which interoceptive predictions and prediction errors shape ‘higher’ levels of selfhood remain exciting areas for investigation [71]. As discussed next, much current evidence in these areas is found in studies of abnormal experiences of selfhood.

## 4. Selfhood and psychopathology

Very generally, the (predictive coding) process theory that we have sketched above for active inference speaks to the synaptic mechanisms that might underlie false inference in psychiatric conditions: in brief, the formal constraints implicit in predictive coding require a modulatory gain control on ascending prediction errors. A recent paper [72] exemplifies how one can understand functional (hysterical) symptoms as false inference about the causes of abnormal sensations, movements or their absence. This example offers a simple (neurophysiological) explanation of symptomatology that is otherwise rather difficult to diagnose or formulate. This theme is emerging repeatedly in psychiatry: from false inference as an account of positive symptoms (hallucinations and delusions) in schizophrenia [73], to the loss of central coherence in autism [74]. Moreover, it is remarkable that the same role for precision-weighting of prediction errors emerges from different theoretical treatments of learning and inference in the brain—including predictive coding in vision [20], free-energy accounts of perception and behaviour [4] and hierarchical Bayesian models of learning [75].

### (a) Autism and interoceptive inference

Perhaps the best example of applying concepts from interoceptive inference to understanding disorders of selfhood can be found in autism research. Recently, much of the phenomenology of autism has been cast in terms of false inference that results from a loss of prior precision, relative to sensory precision [74,76,77]. However, in autism the consequences of increases in (or a failure to attenuate) sensory precision are also being considered in a developmental context, in which one has to accommodate the consequences for acquisition or learning of deep generative models. This is particularly interesting in relation to interoceptive inference because it touches on the acquisition of generative models that distinguish between self and other.

One line of thinking here is that a failure to contextualize interoceptive cues, elicited by interactions with the mother, precludes a proper attribution of the agency to the interoceptive consequences of prosocial interactions [78]. In brief, the idea is that a failure to attenuate the precision of interoceptive prediction errors would not only render autistic infants unduly sensitive to interoceptive cues (i.e. autonomic hypersensitivity) but would have profound implications for a sense of self versus other. This follows from the inability to ignore the absence of interoceptive signals associated with nurturing (e.g. breastfeeding) during affiliative interactions with (m)others. In short, the autistic infant could never learn that the nurturing and prosocial (m)other were the same hidden cause or external object [78] (figure 4). This has several interesting implications for attachment, theory of mind, and a lack of central coherence that characterizes the disorder in later life [79]. It also provides an interesting explanation for interoceptive hypersensitivity (cf. an emotional echopraxia) in autism and failure to engage with prosocial (exteroceptive) cues [80]. If this explanation is right, then it provides a clear pointer to abnormalities of (precision) gain control in cortical systems mediating interoceptive inference such as the anterior insular and cingulate cortex [54,81].

Potential interoceptive abnormalities in autism are unlikely to reside at any single level in the interoceptive hierarchy. In a recent study, a comparison of autistic individuals with controls found that autism was associated with (i) reduced objective interoceptive sensitivity, quantified using standard heartbeat detection tasks and (ii) an increased trait interoceptive sensibility, measured using subjective questionnaires, when compared with controls [82]. These results can be interpreted in terms of an increased ‘interoceptive trait prediction error’ (ITPE) in autism; i.e. a larger mismatch between subjective expectations about interoceptive accuracy and objective interoceptive sensitivity. Interestingly, across both autistic individuals and controls, the magnitude of ITPE correlated with self-reported anxiety, recalling the early proposal of Paulus & Stein [83] which associated anxiety with an interoceptive prediction error (though not in a Bayesian framework). One complication that may nuance this view is that autism often co-occurs with alexithymia (difficulties in identifying and describing one's own emotions); a recent study found that atypical interoception was associated with alexithymia not autism, though this study did not specifically consider ITPEs [84]. More generally, the heterogeneous nature of autism may exclude single process explanations and may underlie apparent inconsistencies in the current empirical data (e.g. another recent study [85] found decreased not increased subjective body awareness in autism).

### (b) Depression and fatigue

Beyond autism, interoceptive inference is emerging as a powerful framework within which to understand depression, fatigue and their interactions. Depression exerts a profound impact on quality of life and carries a very high socio-economic cost. Fatigue is a prominent symptom across a variety of disorders and also exacts a high toll on quality and productivity of life. While depression and fatigue encompass a wide range of cognitive, behavioural and physiological aspects, some recent albeit speculative proposals have implicated disrupted interoception in their aetiology.

In one version of this story, peripheral endocrine and immunological changes accompanying or preceding depressive onset lead to persistently imprecise (‘noisy’) interoceptive afferents [9,86]. This in turn leads to lower precision-weighting of (i.e. reduced attention to) ascending interoceptive signals and correspondingly greater reliance on interoceptive priors for maintaining physiological homoeostasis. Given the translation of interoceptive predictions into homeostatic set-points, this process could set up a positive feedback loop in which greater reliance on prior predictions generates increasingly large and unreliable interoceptive prediction errors, which in turn increases the reliance on the now dysfunctional interoceptive predictions. At some point, the ensuing dyshomoeostasis will tip over into fatigue and sickness behaviour that signal the initial stages of depression [9].

In another version of the story [87], while fatigue and depression are still considered as responses to the interoceptive experience of dyshomoeostasis, these now take the form of metacognitive beliefs about the brain's capacity to successfully regulate bodily states (allostatic self-efficacy). Fatigue is proposed to represent an early response to dyshomoeostasis that retains adaptive value (like sickness behaviours in general), while a generalized belief of low allostatic self-efficacy following prolonged (experienced) dyshomoeostasis may trigger depression, in a way that recalls cognitive theories of ‘learned helplessness’ [88]. Both these accounts of depression are supported by the involvement of agranular visceromotor cortices in the pathophysiology of depression (e.g. [89]). To further refine, distinguish and empirically test these formulations may require advanced model-based neuroimaging analyses—of the sort being developed under the rubric of ‘computational psychiatry’ [90–92].

## 5. Concluding remarks

Applying the framework of active inference to interoception provides a powerful set of concepts within which to conceive the neurofunctional basis of emotion, embodied selfhood and allostatic control. The main points can be summarized as follows. Interoceptive inference parallels other applications of active inference (or prediction error minimization) in proposing that sensory areas convey ascending prediction errors that are compared with descending predictions across a hierarchy of perceptual processing. For interoceptive inference, predictions issue from (agranular) VMAs and project to viscerosensory areas (to provide corollary feedback) as well as to brainstem and subcortical areas (to engage autonomic homoeostatic reflexes). Importantly, visceromotor predictions are best interpreted as providing homoeostatic set-points that enslave autonomic reflexes and guide allostatic (behavioural and physiological) responses via interoceptive prediction errors at different hierarchical levels and timescales. This perspective emphasizes the anticipatory control-oriented nature of interoceptive inference [7], recalling the role of predictive models in cybernetic theories of regulation [14,15] as well as their counterparts in (exteroceptive) perception, e.g. perceptual control theory [18,93].

Mapping the computational architecture of interoceptive inference to neuroanatomical substrates—and considering the key role of precision-weighting—provides the tools to connect these ideas to (i) theories of emotion and embodied selfhood and their experimental manipulation, and (ii) a range of clinical conditions which express interoceptive symptoms and/or plausibly originate via disruptions in interoceptive inference. In terms of theoretical implications, emotional feeling states can be seen as the joint content of interoceptive predictions, while embodied selfhood rests on the multimodal and amodal predictions that distinguish self-related from non-self signals via active inference. Accumulating clinical data and experimental evidence are revealing the mechanisms by which interoceptive signalling shapes experiences of self, and also of perceptions of stimuli originating from the external environment (e.g. [94,95]). However, uncovering empirical evidence that speaks directly in favour of (or against) interoceptive inference stands as an important challenge. Key predictions of the framework are that (i) descending signals from VMAs carry predictions about the causes of interoceptive signals (and, further, that in doing so they serve as homoeostatic set-points), (ii) ascending signals targeting VMAs convey interoceptive prediction errors, and (iii) emotional or affective contents depend primarily on interoceptive predictions rather than prediction errors. Future research could test these predictions using advanced laminar fMRI methods to potentially distinguish ‘prediction’ from ‘prediction error’ responses [31], or by capitalizing on natural variability in physiological rhythms (e.g. heartbeat variability) to model ongoing interoceptive prediction errors that might be reflected in electrophysiological signals (Klaas Enno Stephan 2016, personal communication; see also [96]). Microneurography techniques—which allow direct recording of peripheral nerve traffic [97]—might also provide an innovative means of isolating interoceptive prediction and prediction error signals.

Extending active inference to include autonomic reflexes and interoceptive predictions raises many further interesting questions [26]. For example, can the putative role of neuromodulators (e.g. dopamine and oxytocin) in mediating the precision of prediction errors help to explain the close relationship between arousal and anxiety? What is the relationship between exteroception and interoception during self-observation and how does this depend upon the attenuation of the precision of respective prediction errors [98]? Do von Economo cells in infragranular cortical layers convey interoceptive predictions from the insular cortex to the amygdala and other subcortical targets [99]? How does the control-oriented nature of interoceptive inference shape the qualitative aspects of interoceptive experience, and what in general determines the conscious status of interoceptive predictions? Key questions about hierarchical inference and the role of interoception are also being addressed in the new field of neuropsychoanalysis [100].

The practical implications of these ideas are highlighted by their application to a variety of clinical conditions in which atypical interoceptive inference may play important roles in aetiology and/or in symptom expression. Emotional disorders like alexithymia are relatively straightforward to explain in terms of atypical interoception, while more complex and heterogeneous constructs like anxiety have been considered in terms of interoceptive prediction error for more than a decade [53,83,101]. Recent developments have focused on depression and fatigue as emerging from the interoceptive experience of chronic dyshomoeostasis, whether directly or via metacognitive beliefs in inadequate allostatic self-efficacy [9,87]. Autism, also highly heterogeneous, seems to have a common interoceptive foundation, possibly with a developmental origin and symptom expression characterized by discrepancies between (reduced) objective interoceptive sensitivity and (enhanced) self-appraisal of interoceptive ability. Importantly, the involvement of interoceptive inference in these and other conditions—including, for instance, depersonalization disorder (see [102])—opens new avenues for diagnosis through physiological measures and computational psychiatry approaches, and potential clinical intervention via interoceptive training and feedback.

Altogether, considering embodied selfhood through the lens of prediction error minimization brings a new way to think about an old doctrine. Rene Descartes, besides dividing the world into *res cogitans* and *res extensa*, also achieved a certain notoriety for introducing the doctrine of the ‘beast machine’ (*ca* 1694). He argued that while humans had minds directing their behaviour, non-human animals (‘brutes’) were nothing more than unthinking, unfeeling machines that breathe, digest, perceive and move ‘like clockwork’. Now that we can see how human minds are deeply grounded in embodied physiology, and that similar functional principles may unite physiological regulation with perception of the external world and the guidance of actions and behaviour, an inversion of Descartes’ doctrine seems plausible: that our subjective experiences of selfhood may arise *because of*, and not *in spite of*, the fact that we too are ‘beast machines’.
